# Use of Almond Shells and Rice Husk as Fillers of Poly(Methyl Methacrylate) (PMMA) Composites

**DOI:** 10.3390/ma10080872

**Published:** 2017-07-28

**Authors:** Alessandra Sabbatini, Silvia Lanari, Carlo Santulli, Claudio Pettinari

**Affiliations:** 1 General and Inorganic Chemistry Unit, School of Pharmacy, Università degli Studi di Camerino, via S. Agostino 1, 62032 Camerino (MC), Italy; alessandra.sabbatini@unicam.it (A.S.); claudio.pettinari@unicam.it (C.P.); 2 Teuco S.p.A., via Virgilio Guzzini 2, 62010 Montelupone (MC), Italy; silvia.lanari@teuco.it; 3 School of Architecture and Design, Università degli Studi di Camerino, viale della Rimembranza, 63100 Ascoli Piceno, Italy

**Keywords:** PMMA, almond shells, rice husk, mechanical characterization, viscosity measurements

## Abstract

In recent years, wood fibres have often been applied as the reinforcement of thermoplastic materials, such as polypropylene, whereas their use in combination with thermosetting resin has been less widespread. This study concerns the production of PMMA-based composites by partly replacing alumina trihydrate (ATH) with wood waste fillers, namely rice husks and almond shells, which would otherwise be disposed by incineration. The amount of filler introduced was limited to 10% as regards rice husks and 10 or 15% almond shells, since indications provided by reactivity tests and viscosity measurements did not suggest the feasibility of total replacement of ATH. As a matter of fact, the introduction of these contents of wood waste filler in PMMA-based composite did not result in any significant deterioration of its mechanical properties (Charpy impact, Rockwell M hardness and flexural performance). Some reduction of these properties was only observed in the case of introduction of 15% almond shells. A further issue concerned the yellowing of the organic filler under exposure to UV light. On the other hand, a very limited amount of water was absorbed, never exceeding values around 0.6%, despite the significant porosity revealed by the filler’s microscopic evaluation. These results are particularly interesting in view of the application envisaged for these composites, i.e., wood replacement boards.

## 1. Introduction

In recent years, disposing of agro-waste in ways other than incineration or landfilling has often been considered. In particular, when agro-waste are wood-like materials, hence with a significant lignin content, they may be introduced in a material (most often in polymers) and modify, hopefully in a positive sense, its mechanical and structural performance.

As regards the materials that are considered in this study, rice hulls have been proposed as the filler for cement-based composites [1]. Rice hulls can be considered as roughly formed by around 40 wt % cellulose, 30 wt % lignin, 20 wt % silica and 10 wt % other components [2]. The combined presence of lignin and silica in the porous structure of rice hulls shows potential for example for the decontamination of environmental objects [3], while on the other hand providing significant interest in terms of offering an increased hardness and tear strength to a polymer matrix [4]. It is worth considering though that an increase of properties by the introduction of rice husk in materials is generally obtained through the extensive use of modifications, such as grafting [5] or application of foaming agents [6]. A work on poly(methyl methacrylate) with rice husk reported the application of a treatment with triphenylphosphine [7].

In contrast, almond shells are composed of a 37 wt % of cellulose, 32 wt % hemicellulose, 27 wt % of lignin and 4 wt % of other components [8]. Most studies are aimed therefore at their pyrolysis, in some cases discussing also the enzymatic digestibility of their cellulose content [9]. However, attempts in the sense of their re-use as filler in materials have been limited so far. It is promising though that their introduction in amounts up to 30 wt % in a polypropylene matrix without treatment improved the stiffness of the composite by over 50%, demonstrating also a sufficient compatibility with the matrix and increasing the temperatures for the onset of thermal decomposition [10]. Another study concerned their introduction in a biodegradable polymer, such as polylactic acid (PLA), in which case it was interestingly suggested that thermal decomposition behaviour of almond shell does not substantially depend on the almond variety, which means that inherent variability of the agro-waste filler does not result in significantly affecting injectability [11].

This study is aimed at evaluating the introduction of the above fillers, namely rice husk and almond shells, to compare their effectiveness in partly replacing alumina trihydrate (ATH) in poly(methyl methacrylate) matrix (PMMA). This is a common practice when PMMA is used as a replacement material for wood, which may for example improve its fatigue performance [12] or compensate for its rather poor deformability and processability [13]. This has a particular significance, since ATH derives from the industrial treatment of bauxite (a mineral obtained by mining) with sodium hydroxide at 270 °C [14], and also because the introduction of fillers, such as rice husk or almond shells, does result in some weight reduction (ATH density is around 2.4, against around 0.6 for almond shells [15] and around 0.55 for rice husk [16]). The objective would also be some cost reduction, considering that ATH, when ordered on large scale, has a market price in the order of 1000 Euros per ton, while almond shells or rice husk would come from the food processing system at basically no cost. The main question and hence the objective of this work is to elucidate whether this addition does not compromise processing and mechanical performance, so to allow the application of the PMMA-based material as a wood replacement board.

## 2. Experimental

### 2.1. Preliminary Work: Reactivity Tests

**Figure 1:**
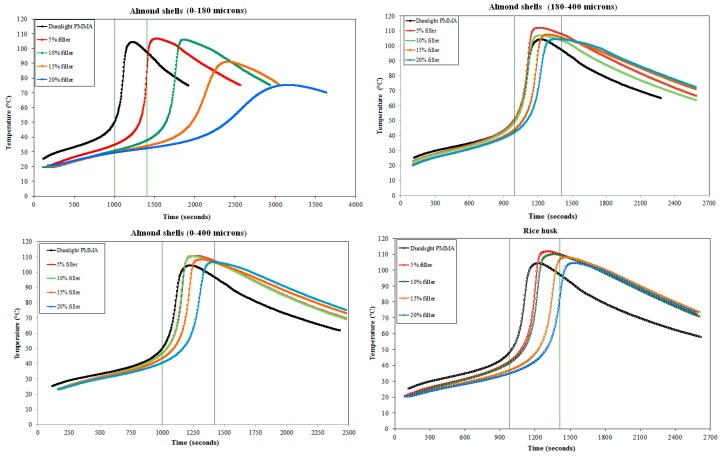
Temperature vs. time polymerization curves for different fillers in different amounts (the two vertical lines highlight the range of exothermic peak, which offers the optimal behaviour).

The exothermic polymerization was recorded by a thermocouple data logger, PicoLog D-08, on the reference material (Duralight^®^ PMMA) and on the material with added fillers, ground almond shells with granulometries 0–180 μm, 180–400 μm and 0–400 μm and rice husk with granulometry 0–400 μm, in amounts from 5 to 20%. The thermocouple is positioned in the centre of the pot which contains the slurry. Data logging is started when the thermocouple is immersed in the slurry and the time is counted starting from the addition of the last catalyst. The acquisitions are performed every 5 s and the registration is stopped when the polymerization temperature becomes lower than 75 °C. The optimal formulations are those having the exothermic peak in the time range 1200 (±200) s, characteristic of Duralight^®^ with 62 wt % filler, a range which is highlighted by vertical lines in Figure 1.

### 2.2. Preliminary Work: Viscosity Measurement

The viscosity measurements were performed by using a Brookfield DV-II+ viscometer (AMETEK Brookfield, Middleboro, MA USA), at different spindle rates, namely 2, 4 and 10 revolutions per minute (RPM). The spindle rates are chosen in order to verify the organic filler influence on the pseudoplastic behaviour characteristic of PMMA resin. Two different spindle typologies were used, LV3 and LV4. The different spindles are used when the torque percentage is out of 100%, resulting in a not detected value of viscosity. The viscometer has conversion factors which make comparable the viscosity values obtained by the different spindles. The reference value is considered that obtained at 10 RPM with a torque percentage in the range 5–100%. The acrylic slurries have a temperature of 21 ± 2 °C and the accuracy of the instrument is ±1% full scale rang in use. The viscosity measurement is performed to characterize the dispersion prior to carry out the production of PMMA sheets, in the understanding that higher viscosities would lead to air entrapment and formation of air bubbles, thus affecting the mechanical properties of the material.

### 2.3. Materials and Formulations Selected for Testing

Duralight^®^ PMMA contains, as reported in a relevant patent [17], an ATH content in the order of 50–70 wt %, according to the required workability of the material. A total amount of 62 wt % of filler has been selected for this study, which is close to average ATH content in Duralight^®^. The introduction of organic fillers (rice husk or ground almond shells) resulted obviously in the reduction of ATH content in the material. The optimal percentage of rice husk and ground almond shell is established by combing the reactivity test (Figure 1) and viscosity measurements (Table 1) of all dispersions (5–20 wt % of waste filler). In practice, four different types of materials were finally developed, as presented in Table 2. One formulation (F1) includes rice husk, while other three (F2 to F4) include almond shells with different granulometries.

### 2.4. Mechanical Tests

Three point flexural tests according to UNI EN ISO 178 standard were performed using a Galdabini Quasar 10 dynamometer (Galdabini, Cardano al Campo (VA), Italy). After an automatic pre-load of 2 N, the load was applied in displacement control mode and employing a cross-head speed of 2 mm/min on specimens with dimensions 180 mm length, 15 ± 0.5 mm width and 9.5 ± 0.5 mm thickness. The span length used was 160 mm.

Hardness measurement (Rockwell type M) was performed using an OMAG 206 RTD durometer (Casavola Macchine Utensili S.p.A., Cinisello Balsamo (MI), Italy) according to UNI EN ISO 2039-2 standard. After a pre-load of 98.07 N ± 2%, a load of 980.70 N ± 2% was applied on specimens with minimum thickness of 6 mm. 

Charpy impact tests were performed according to UNI EN ISO 179-1 standard, using an “Impact Plast” Galdabini (Galdabini, Cardano al Campo (VA), Italy) pendulum with energy of 1 J, parallel to the thickness, on unnotched specimens with minimum length of 80 ± 2 mm, minimum width of 10.0 ± 0.2 mm and minimum thickness of 4.0 ± 0.2 mm. The span length was 62 mm.

### 2.5. UV Resistance 

UV resistance at dry conditions was examined by exposure of the sample surface to nine OSRAM Ultra-Vitalux 300 W lamps (OSRAM SpA, Milano, Italy). The change in colour to be attributed to UV weathering was evaluated using an X-Rite Ci62 colorimeter (X-Rite, Grand Rapids, MI, USA) after 250 and 500 h of exposure. In particular, the CIE/Lab L*a*b colour space tristimulus values and related quantities allows eliminating subjectivity in colour perceptions and colour difference judgments [18]. In the L*a*b* diagram, a spherical colour solid, L* indicates lightness, and a* and b* are the chromaticity coordinates. Here, a* and b* indicate colour directions (+a* is the red direction, −a* is the green direction while +b* is the yellow direction and the −b* is the blue direction). By comparing measurements of target colours with sample specimens, it is possible to express the nature of a colour difference between two measured specimens. 

### 2.6. Resistance to Water 

Tests to evaluate the resistance to boiling water (immersion for 2 h) and to steam (exposure for 1.5 h) were performed by using an SB FALC thermostatic bath (FALC INSTRUMENTS SRL, Treviglio (BG), Italy) at 100 °C, measuring once again colour variation with an X-Rite Ci62 colorimeter (X-Rite, Grand Rapids, MI, USA). 

Water absorption measurements were carried out according to UNI EN ISO 62 standard and the sample mass was measured using an Orma EB200S analytic balance (Orma Srl, Milano, Italy). 

### 2.7. Scanning Electron Microscopy

Scanning electron microscopy (SEM) for the observation of sectioned samples was carried out using a Cambridge Stereoscan S260 microscope (CAMBRIDGE SCIENTIFIC PRODUCTS, Watertown, MA, USA). To allow their observation, the samples were gold coated.

## 3. Results

The MMA polymerization is a free radical reaction, characterized by an exothermic peak during the process. The amount of filler introduced in the formulation influences the exothermic peak both in time and temperature. The study of the polymerization curve is necessary to establish the right amount of organic filler that can be introduced in the formulation to replace ATH, avoiding modifications in the exothermic curve compared to that of Duralight^®^. In fact, a lower temperature peak and/or a delay result in an incomplete reaction in the material and, as a consequence, the presence of a higher porosity and therefore possibly higher absorption of liquids and lower physical and mechanical properties, which is well known on PMMA [19,20]. From the temperature vs. time curves reported in Figure 1 the following evidences can be outlined:

(1) Keeping constant the catalysts amount, the comparison of the rice husk exothermic peaks with the standard Duralight^®^, makes visible that the reactivity peak obtained with 5 and 10% of rice husk is located in the reactivity range of Duralight^®^. In order to substitute the highest amount of ATH with the organic filler, the reference formulation contains a 10% of rice husk.
(2) A comparison of the almond shell (0–180 μm) dispersion exothermic peaks with the standard Duralight^®^, makes visible that the reactivity peak is not reached in the reactivity range of Duralight^®^. Concerning the temperature, the compliant formulations are those containing 5 and 10% of almond shell (0–180 μm) replacing ATH filler. Hence, the reference formulation contains 10% of almond shells (0–180 μm).
(3) A comparison of the almond shell (180–400 μm) dispersion exothermic peaks with the standard Duralight^®^, makes visible that all the reactivity peaks are compliant in time and temperature. The reference formulation will contain 15% of almond shells, instead of the 20%, which itself complies to Duralight^®^ standard, because the dispersion with 20% of almond shell has a highly variable viscosity and therefore is likely to have not very consistent properties. This fact can bring to formation of defects during the sheets production (i.e., air bubbles) leading to invalid mechanical characterization.
(4) A comparison of the almond shell (0–400 μm) dispersion exothermic peaks with the standard Duralight^®^, makes visible that the reactivity peaks obtained with 5, 10 and 15% of almond shells (0–400 μm) are compliant in time and temperature. The reference formulation will contain 10% of almond shells, instead of the 15 or 20%, which themselves comply to Duralight^®^ standard, because the dispersions have high viscosity.

The viscosity measurements of all formulations are reported in Table 1 and show generally a decreasing viscosity with increasing shear rate. This means that the dispersions have a pseudoplastic behaviour as shown by Duralight^®^. This type of flow behaviour is sometimes called “shear-thinning” [21]. Viscosity needs to be low enough to avoid the air entrapment and the consequent formation of defects. The maximum viscosity values, at 10 RPM, for the hand-workability of the dispersions, were measured as 12,297, 20,575, 9586 and 11,313 cPs for F1, F2, F3 and F4, respectively.

**Figure 2:**
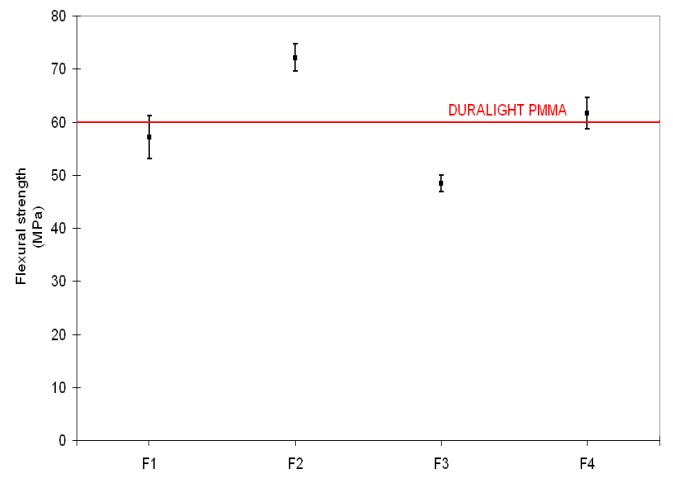
Flexural strength results.

**Figure 3:**
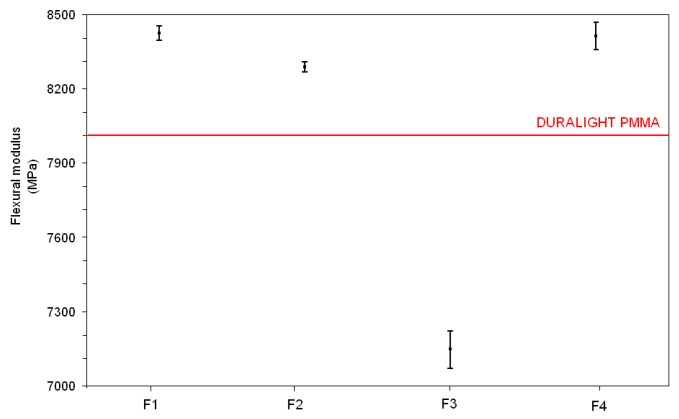
Flexural modulus results.

**Figure 4:**
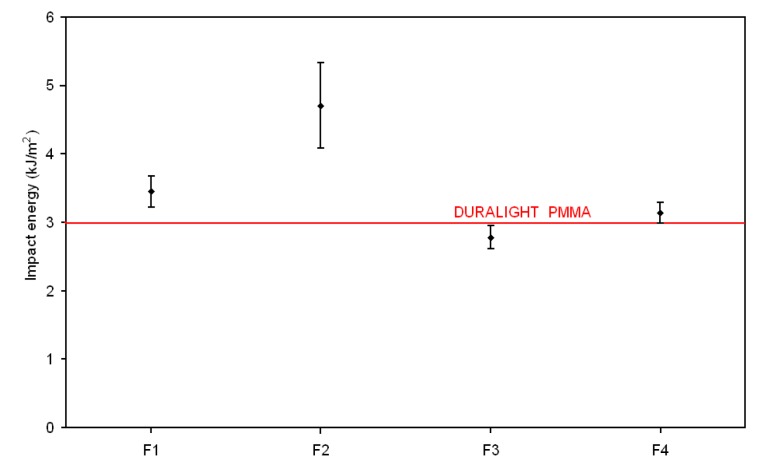
Charpy impact results.

**Figure 5:**
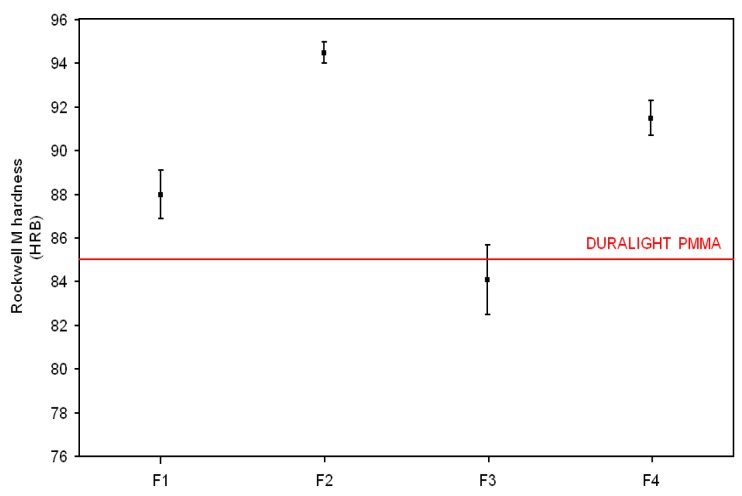
Rockwell hardness results.

Mechanical properties (flexural strength and stiffness, Charpy impact and Rockwell hardness), reported in Figure 2, Figure 3, Figure 4 and Figure 5, respectively, have been examined against the values declared in the specifications for the Duralight^®^ composite material. As it was expected from reactivity data, three out of four configurations, namely F1, F2 and F4, are basically not affected by the filler addition in terms of strength and show improved performance in all other parameters i.e., stiffness, Charpy impact and Rockwell hardness. In contrast, a significantly lower performance is revealed by the F3 formulation, which shows both flexural strength and stiffness reduced with respect to Duralight^®^ by around 15%, whilst very limited decreases have been revealed as regards impact and hardness values. This deceiving performance can be therefore attributed to the presence of large fragments of almond shells, which have limited compatibility with the polymer matrix. This may be expected, as the introduction of material from nutshells in polymers is usually realised in the form of ground powder, therefore not exceeding a few microns size [22].

Samples exposed to UV light show a yellowing of the organic filler, in particular in the case of rice husk, hence for F1 formulation, where a positive Δb data is observed, as from Table 3. The yellowing process is natural for wood materials exposed to UV because of the oxidation reaction of the cellulose. The photolysis reaction involves the primary hydroxyl group on the C6 and the secondary hydroxyl groups on the C2 and C3 of the cellulose molecules. These reactions cause darkening and yellowing phenomenon [23]. In the case of almond shells, F2, F3 and F4, the yellowing effect is less consistent, probably due to the lower content of cellulose and to the darker initial colour of the samples.

**Figure 6:**
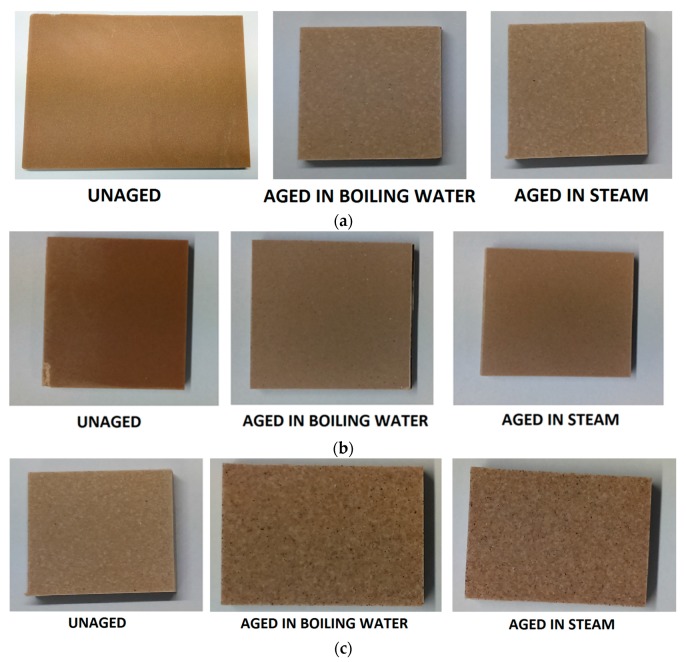

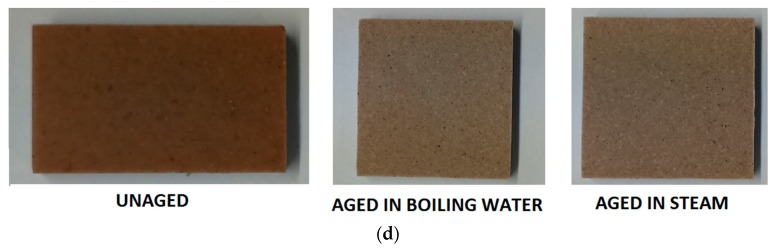
(**a**) Colour change due to ageing in formulation F1; (**b**) Colour change due to ageing in formulation F2; (**c**) Colour change due to ageing in formulation F3; (**d**) Colour change due to ageing in formulation F4.

**Figure 7:**
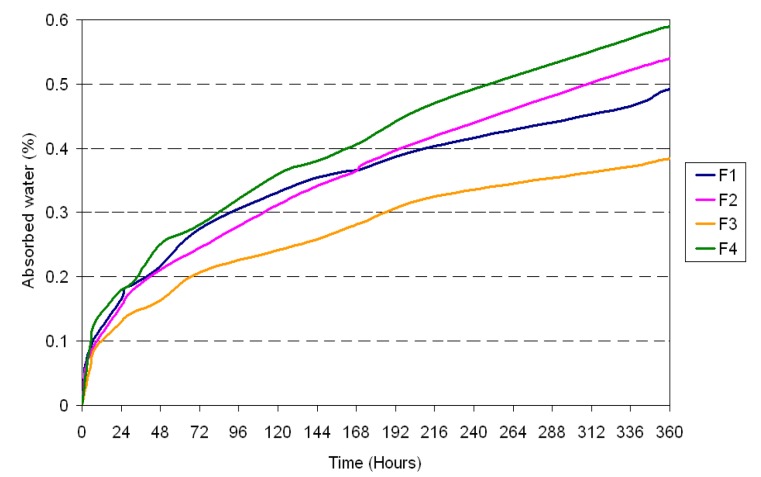
Absorbed water after immersion at ambient temperature.

On the other hand, samples tested in boiling water (2 h) and in contact with steam (1.5 h) show a serious change in colour relative to the standard samples, which are inherently translucent: a global representation of the colour of samples in the different conditions is reported in Figure 6a–d. In particular, a whitening process is more likely to be observed. The likely explanation of this phenomenon is that micro-fissures are formed between the polymeric matrix and the inorganic and organic fillers: this phenomenon has been frequently observed in the interaction between PMMA and ATH for example in architectural panels [24]. In the present case, despite the composite surface being slightly rough, no filler loss has been detected in any samples under the ageing conditions investigated. Furthermore, the amount of wood filler introduced does result in the absorption of just a minimal amount of water (Figure 7).

**Figure 8:**
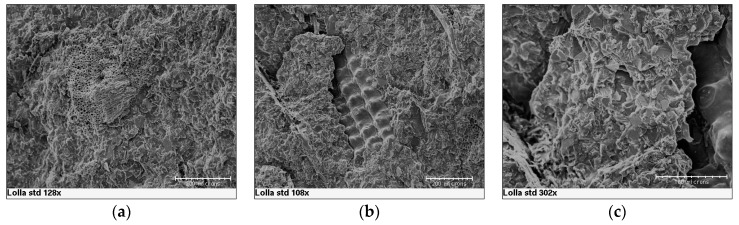
(**a**–**c**) SEM micrographs showing the interaction of rice husk within the poly(methyl methacrylate) matrix- alumina trihydrate (PMMA-ATH) matrix.

**Figure 9:**
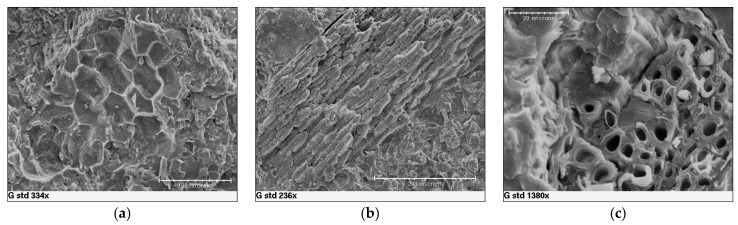
(**a**–**c**) SEM micrographs showing the interaction of almond ground shells within the PMMA-ATH matrix.

SEM observations were conducted in order to study the interaction existence between the polymeric matrix and the organic filler. In the case of rice husk, it is possible to observe that the filler has a tubular structure and it is disposed linearly (Figure 8a) or in bundles (Figure 8b). The rice husk filler is very well wrapped in the polymeric matrix even if, in some cases, a detachment is detected (Figure 8c). This can be explained by the fact some weak interactions as hydrogen bonds exist, but there is not a reaction between the matrix and the filler which can create a strong chemical bond. However, there is no evidence of filler particles clearly pulling out of the matrix, in contrast with what indicated by a previous study on polypropylene/rice husk composites [25]: this could also be attributed to the lower amount of filler introduced in our case. As a consequence, the composite has a heterogeneous morphology which is responsible for the decreased mechanical properties, compared to the Duralight^®^ values. Conversely, in the case of almond shells, it is possible to observe that the filler has a honeycomb (Figure 9a) and tubular structure (Figure 9b). The almond shells are well covered by the polymeric matrix, but a neat separation is observed at the interface between the matrix and the filler (Figure 9c). As for rice husk, its detachment from the matrix creates a heterogeneous structure, which in turn decreases the mechanical resistance, and in this case a clearer pull-out is observable than it was the case for rice husk. Some occurrence of pull-out has been explained by the fact that the thermoplastic used is not a compatibilized one, such as maleic anhydride polypropylene (MAPP), which as a matter of fact provided better properties for wood-plastic composites [26]. On the other hand, application of ground almond shells as bio-waste resource in thermosetting (e.g., urea-formaldehyde) polymer panels for wood replacement proved effective [27]: yet the matrix is not recyclable in that case, while both PMMA and MAPP would be. In general terms, the introduction of fillers, as rice husk and almond shells, would be regarded as possible and a viable option in production of PMMA polymer panels, although some caution would be needed in terms of increasing their amounts above 10%, in that this would possibly compromise material production.

## 4. Conclusions

The replacement of ATH filler in PMMA-based composite materials by organic bio-waste filler, in particular rice husk or almond shells, is possible up to an amount of 15% in weight. However, reaching effective results would depend from the morphology and the distribution of the filler itself, obviously with more criticality the higher its amount. 

After analysing the results, it can be concluded that:

All the agricultural waste filler allows the polymerization of the composite materials.

The laboratory sheet has good mechanical properties compared to Duralight^®^ standard, in particular no real pull-out is observed from the matrix and the decrease in flexural, impact and hardness properties is far from being critical.

Tests in boiling water and in contact with steam reveal some incompatibility of the acrylic resin with the fillers, which leads to some critical whitening effect. The UV exposure shows a yellowing of the organic recycled fillers, namely in the case of rice husk that is due to the natural photo-oxidation of cellulose.

As a final consideration, this strategy can be considered viable on PMMA composites, though with some issues in terms of filler distribution and amount to be introduced and also in terms of behaviour over time. Notwithstanding that, it is suggested that the use of compatibilizers may improve this picture, possibly leading to successful reformulation of the composite with even larger amounts of bio-waste fillers and therefore with some further environmental benefits.

## Figures and Tables

**Table 1:** Viscosity measurements (at a temperature of 21 ± 2 °C) for different fillers and amounts.

**Rice Husk 0–400 μm**	**Spindle**	**Viscosity (cPs)**			**Temperature (°C)**	**% Torque min**	**% Torque max**	
		**2 RPM**	**4 RPM**	**10 RPM**				
5%	LV3	8578	8458	8182	20.6	14.3	68.2	
10%	LV4	14397	13197	12297	19.0	4.8	20.5	F1
15%	LV4	30293	26994	22255	20.1	10.1	37.1	
20%	LV4	48889	37342	29813	21.5	16.3	49.7	
**Almond Shell 0–180 μm**	**Spindle**	**Viscosity (cPs)**			**Temperature (°C)**	**% Torque min**	**% Torque max**	
		**2 RPM**	**4 RPM**	**10 RPM**			
5%	LV4	15597	14097	12237	20.5	5.2	20.4	
10%	LV4	29394	24745	20576	19.7	9.8	34.3	F2
15%	LV4	58487	46640	35272	21.2	19.5	58.8	
20%	LV4	107377	84731	n. d.	21.4	35.8	56.5	
**Almond Shell 0–400 μm**	**Spindle**	**Viscosity (cPs)**			**Temperature (°C)**	**% Torque min**	**% Torque max**	
		**2 RPM**	**4 RPM**	**10 RPM**			
5%	LV3	10138	9298	8146	21.5	16.9	67.9	
10%	LV3	14217	12927	11313	22.3	23.7	94.3	F3
15%	LV4	25195	21145	17156	22.1	8.4	28.6	
20%	LV4	32093	27864	23694	21.3	10.7	39.5	
**Almond Shell 180–400 μm**	**Spindle**	**Viscosity (cPs)**			**Temperature (°C)**	**% Torque min**	**% Torque max**	
		**2 RPM**	**4 RPM**	**10 RPM**			
5%	LV3	9778	9088	8182	20.4	16.3	68.2	
10%	LV3	9538	8728	8254	20.3	15.9	68.8	
15%	LV3	12777	10888	9586	20.8	21.3	79.9	F4
20%	LV4	12597	10798	9478	22.1	4.2	15.8	

**Table 2:** Composition of the four material formulations produced.

Component	F1 (%)	F2 (%)	F3 (%)	F4 (%)
Acrylic resin	36.35	36.35	36.35	36.35
Pre-treated ATH (20 µm grade)	52	52	47	52
Rice husk 0–400 μm	10			
Almond shell (0–180 μm)		10		5
Almond shell (180–400 μm)			15	5
Peroxide	0.45	0.45	0.45	0.45
Accelerator	0.2	0.2	0.2	0.2
Activator	1	1	1	1

**Table 3:** Colour change due to UV weathering.

Exposure		F1	F2	F3	F4
250 h	ΔL	0.86	−0.47	0.54	0.51
	Δa	−0.16	0.14	−0.61	−0.38
	Δb	1.24	−0.28	0.72	−0.45
	ΔE	1.52	0.56	1.08	0.78
500 h	ΔL	1.70	−0.25	1.64	1.81
	Δa	−0.20	0.46	−1.13	−0.76
	Δb	1.21	0.23	−0.40	−0.71
	ΔE	2.10	0.57	2.03	2.08

## References

[1] He J., Jie Y., Zhang J., Yu Y., Zhang G. (2013). Synthesis and characterization of red mud and rice husk ash-based geopolymer composites. Cem. Concr. Compos..

[2] Chindaprasirt P., Homwuttiwong S., Jaturapitakkul C. (2007). Strength and water permeability of concrete containing palm oil fuel ash and rice husk-bark ash. Constr. Build. Mater..

[3] Qu Y., Tian Y., Zou B., Zhang J., Zheng Y., Wang L., Li Y., Rong C., Wang Z. (2010). A novel mesoporous lignin/silica hybrid from rice husk produced by a sol–gel method. Bioresour. Technol..

[4] Ishak Z.A.M., Bakar A.A. (1995). An investigation on the potential of rice husk ash as fillers for epoxidized natural rubber (ENR). Eur. Polym. J..

[5] Morsi S.M., Pakzad A., Amin A., Yassar R.S., Heiden P.A. (2011). Chemical and nanomechanical analysis of rice husk modified by ATRP-grafted oligomer. J. Colloid Interface Sci..

[6] Manap N.R.A., Jais U.S. (2009). Influence of concentration of pore forming agent on porosity of SiO_2_ ceramic from rice husk ash. Mater. Res. Innov..

[7] Arora S., Mahender K., Mahesh K. (2013). Preparation and thermal stability of poly(methyl methacrylate)/rice husk silica/triphenylphosphine nanocomposites: Assessment of degradation mechanism using model-free kinetics. J. Compos. Mater..

[8] Caballero J.A., Conesa J.A., Font R., Marcilla A. (1997). Pyrolysis kinetics of almond shells and olive stones considering their organic fractions. J. Anal. Appl. Pyrolysis.

[9] Martínez J.M., Reguant J., Montero M.A., Montané D., Salvadó J., Farriol X. (1997). Hydrolytic pretreatment of softwood and almond shells. Degree of polymerization and enzymatic digestibility of the cellulose fraction. Ind. Eng. Chem. Res..

[10] Essabir H., Nekhlaoui S., Malha M., Bensalah M.O., Arrakhiz F.Z., Qaiss A., Bouhfid R. (2013). Bio-composites based on polypropylene reinforced with almond shells particles: Mechanical and thermal properties. Mater. Des..

[11] García A.M., García A.I., Cabezas M.A.L., Reche A.S. (2015). Study of the influence of the almond variety in the properties of injected parts with biodegradable almond shell based masterbatches. Waste Biomass Valoriz..

[12] Kyziol L. (2014). Fatigue strength of wood polymer composite. J. KONES.

[13] Seki M., Tanaka S., Miki T., Shigematsu I., Kanayama K. (2016). Extrudability of solid wood by acetylation and in-situ polymerisation of methyl methacrylate. BioResources.

[14] Rothon R.N. (2001). Particulate fillers for polymers. Rapra Rev. Rep..

[15] Gao M., Tang J., Johnson J.A., Wang S. (2012). Dielectric properties of ground almond shells in the development of radio frequency and microwave pasteurization. J. Food Eng..

[16] Yalçin N., Sevinç V. (2001). Studies on silica obtained from rice husk. Ceram. Int..

[17] TEUCO GUZZINI S.p.A. Montelupone (IT) (2012). Process for Making a Composite Material. European Patent.

[18] McLaren K. (1976). The development of the CIE 1976 (L* a* b*) uniform colour space and colour-difference formula. Colouration Technol..

[19] Kostić M., Petrović M., Krunić N., Igić M., Janošević P. (2014). Comparative analysis of water sorption by different acrylic materials. Acta Med. Median..

[20] Shamoud A.M. (2002). The influence of humidity on the deformation and fracture behaviour of PMMA. J. Mater. Process. Technol..

[21] Mezger T.G. (2002). The Rheology Handbook.

[22] Sutivisedsak N., Cheng H.N., Burks C.S., Johnson J.A., Siegel J.P., Civerolo E.L., Biswas A. (2012). Use of nutshells as fillers in polymer composites. J. Polym. Environ..

[23] Müller U., Rätzsch M., Schwanninger M., Steiner M., Zöbl H. (2003). Yellowing and IR-changes of spruce wood as result of UV-irradiation. J. Photochem. Photobiol. B.

[24] Byrdy A., Kołaczkowski M. (2015). Environmental impacts on the strength parameters of mineral-acrylic (PMMA/ATH) facade panels. Int. J. Polym. Sci..

[25] Yang H.-S., Kim H.-J., Son J., Park H.-J., Lee B.-J., Hwang T.-S. (2004). Rice-husk flour filled polypropylene composites; mechanical and morphological study. Compos. Struct..

[26] Garcia M., Hidalgo J., Garmendia I., García-Jaca J. (2009). Wood—plastics composites with better fire retardancy and durability performance. Compos. Part A.

[27] Pirayesh H., Khazaeian A. (2012). Using almond (*Prunus amygdalus* L.) shell as a bio-waste resource in wood based composite. Compos. Part B.

